# Peptide-Functionalized Carbon Nanotube Chemiresistors: The Effect of Nanotube Density on Gas Sensing

**DOI:** 10.3390/s23208469

**Published:** 2023-10-14

**Authors:** Daniel Sim, Tiffany Huang, Steve S. Kim

**Affiliations:** 1 Air Force Research Laboratory (AFRL), 711th Human Performance Wing, Wright-Patterson Air Force Base, Dayton, OH 45433, USA; daniel.sim.ctr@us.af.mil (D.S.); tiffany.huang14@gmail.com (T.H.); 2 Integrative Health & Performance Sciences Division, UES Inc., Dayton, OH 45432, USA

**Keywords:** carbon nanotube, nanotube density, gas sensor, biorecognition elements, chemiresistor

## Abstract

Biorecognition element (BRE)-based carbon nanotube (CNT) chemiresistors have tremendous potential to serve as highly sensitive, selective, and power-efficient volatile organic compound (VOC) sensors. While many research groups have studied BRE-functionalized CNTs in material science and device development, little attention has been paid to optimizing CNT density to improve chemiresistor performance. To probe the effect of CNT density on VOC detection, we present the chemiresistor-based sensing results from two peptide-based CNT devices counting more than 60 different individual measurements. We find that a lower CNT density shows a significantly higher noise level and device-to-device variation while exhibiting mildly better sensitivity. Further investigation with SEM images suggests that moderately high CNT density with a stable connection of the nanotube network is desirable to achieve the best signal-to-noise ratio. Our results show an essential design guideline for tuning the nanotube density to provide sensitive and stable chemiresistors.

## 1. Introduction

Wearable sensors monitoring human physiological and physical statuses are emerging as a pivotal component to protect, enhance, and sustain human performance in operational fields. Amongst the human physiological and physical indicators, volatile organic compounds (VOCs) in exhaled breath serve as essential biomarkers to assess human performance [1,2,3,4], such as fatigue level, cognitive decision ability, and physical status. In addition, VOCs are important indicators to assess environmental conditions with an acute impact. Existing VOC sensors are mostly not applicable to wearables due to the lack of meeting the size, weight, and power (SWaP) requirements [5,6,7]. Most commercial VOC sensors are not effective in wearables due to their low selectivity and sensitivity [8,9,10,11]. Many research groups have recently investigated biorecognition elements (BREs) to discover specific sensing materials that are selective and sensitive to target molecules [12,13,14,15,16]. BREs are biological materials such as enzymes, antibodies, and peptides whose chemical properties can be artificially engineered; BREs have a high potential for specific detection of targets of interest by tuning their molecular affinities. In order to efficiently measure events between BREs and target VOCs, it is crucial to implement the appropriate device platform that effectively transduces BREs’ events and provides high-quality measurable signals [17]. Carbon nanotube (CNT) is one of the ideal transducing materials for the BRE interface due to its large surface area [18] and chemical stability [19]. It is also suitable for portable/wearable applications due to its ability to operate continuously [20,21] and fit nano-sized devices [20]. When target molecules come to the BRE-functionalized CNTs, the transducing process occurs by changing CNTs’ electrical properties such as resistance. CNT chemiresistors using the above principle have been widely utilized to detect several biomarkers [13,21,22,23,24,25,26]. In addition, a straightforward CNT post-assembly process (i.e., dielectrophoresis, DEP) has allowed us to build CNT sensors in a more accessible way. The DEP method applies electric fields to the CNT solution to align the CNT network across the electrodes, thus offering a more straightforward pathway to build CNT sensor platforms by minimizing assembly time and CNT contaminations due to its post-process characteristics. However, one of the challenges associated with DEP-based CNT chemiresistors is that their sensing performance is strongly dependent on the density of the CNT network. While several researchers have studied single-strand CNT sensors [27,28] to mitigate the variance in the nanotube density, practical issues have persistently appeared in their low yield rates and device stability. CNT-network-based sensors have shown better reproducibility and durability [27], and are thus being accounted as a more desired platform for practical applications. To investigate the effect of nanotube density, Ishikawa et al. [29] investigated three CNT sensors with different nanotube networks of low, medium, and high density. The above study found that the sensor with low-density CNTs showed better sensitivity (target molecule: streptavidin protein). In contrast, the sensor with high-density CNTs showed a reduced sensitivity of less than 20% of the low-density CNTs. In another previous study [30], Suehiro et al. exposed a constant amount of NO_2_ to CNT sensors with four different nanotube densities with their resistance ranging from 10–100 KΩ. The sensor with the lowest density showed higher sensitivity than that with the highest density. While these studies have shown that CNT density plays a critical role in sensor application, further parametric analyses are still needed to understand the mechanism of nanotube-network-based chemiresistors. For example, a multivariate analysis accounting for not only sensitivity but also noise level and signal-to-noise ratio (SNR) is important in probing the efficacy of sensor operation. Furthermore, testing a large number of devices with a wide range of nanotube densities enables one to probe reliable correlations between nanotube density and sensing performance. There is also a need to characterize BRE-functionalized CNTs, as BREs serve as an important sensing component for enhancing selectivity and sensitivity. We previously reported a preliminary study on the effect of CNT density for peptide-functionalized CNT chemiresistors by investigating sensitivity, noise, and signal-to-noise ratio (SNR) [31]. While the results suggest critical design guidelines for controlling CNT density, further investigation addressing low CNT resistance of less than 1 KΩ and additional experimental tests using other peptides and VOCs have been required to validate the reliable correlation between CNT density and sensor performance.

Here, we present a study on peptide-functionalized CNT chemiresistors to investigate the effect of nanotube density on various sensing parameters. The main idea of this work is to analyze several tens of various chemiresistors with different nanotube densities to comprehensively derive a conclusion on the effect of nanotube density on gas sensing. In our previous publication [32], we addressed the rational design of peptide sequences interfacing with carbon surfaces by analyzing the conformational characteristics of the peptide. This paper compliments the above work on unveiling how the CNT density affects CNT chemiresistor gas-sensing performance. By controlling the CNT assembly process, we fabricate chemiresistors that exhibit widely distributed CNT resistances ranging from 0.5 KΩ to 200 MΩ, presuming that CNT density is inversely proportional to the CNT resistance. We formulate and test more than 30 different chemiresistors with a wide range of nanotube densities for the reliable analysis. In addition, we provide a comprehensive perspective on the effect of nanotube density not only on sensitivity but also on the noise level and SNR (a key indicator of signal quality). While the previous works mainly focused on sensitivity [29,30], this study focuses more on signal quality and device-to-device variability for assessing comprehensive sensor performance.

Here we use short peptides as BREs to functionalize CNTs. The short peptides are promising BREs due to the fact that the peptides can (1) be synthesized using readily available synthesis tools, (2) exhibit diverse physiochemical properties, and (3) be chemically stable. Therefore, peptides can serve as bioreceptors enabling CNT sensors to be more selective and sensitive to target molecules. Due to these characteristics, peptides have widely functionalized CNTs for selective molecular detection [33]. Here, we use two representative peptide BREs: a peptide from the phage display with a high affinity to CNTs [34] and a peptide from molecular dynamics and machine learning [32]. Both peptides are from generally accepted methods (i.e., biological or computational) to predict and optimize peptide sequences, thus representing our current study’s applicability to general peptide-based biorecognition elements. As a comparison to the peptide BREs, we also tested non-peptide based CNT chemiresistors to confirm that this study’s experimental discoveries can be applied to broader ranges of CNT sensor applications as well. The experimental data obtained from the IPA and acetone exposure test suggests that a moderately high nanotube density shows the optimal sensing performance in SNR. This study provides essential information on a CNT network design for optimizing CNT-based VOC sensors.

## 2. Materials and Methods

### 2.1. Materials 

Isopropyl alcohol (IPA) (C_3_H_8_O, 99.5%) and acetone (C_3_H_6_O, ≥99.5%) were purchased from Sigma-Aldrich (St. Louis, MO, USA). Carbon nanotube powder (small diameter SWNTs, HiPcoTM) was purchased from NanoIntegris, Inc. (Skokie, IL, USA). All peptide sequences (with a purity of 90%) were purchased from Peptide 2.0 Inc. (Chantilly, VA, USA). Distilled water (UltraPure™ DNase/Rnase-Free Distilled Water) was purchased from Life Technologies (Carlsbad, CA, USA).

### 2.2. Chemiresistor Platform Fabrication

The fabrication for the chemiresistor platform is based on the previous process [32]. Briefly, a 4-inch silicon wafer with a 0.2 µm-thick SiO_2_ layer was used following a cleaning process. The conventional photolithography process was performed using photoresists of LOR10A and AZ514 and mask-based ultraviolet light exposure to pattern electrode pair configurations. Then, the 15 nm/65 nm thickness of the Ti/Au layer was thermally evaporated onto the wafer, followed by a lift-off process. The individual chemiresistor set is completed by dicing the wafer into the total dimensions of the platform containing nine pairs of electrodes.

### 2.3. Peptide-Functionalized CNT Solution and Assembly

A peptide-functionalized CNT solution preparation consisting of 150 µL of a peptide solution (1.5 mg peptide (either FYYYLLQ (P1) or GSVQKLSATPWV (P2)) + 150 µL water), 1.5 mg of a CNT powder, and 1.35 mL of water were added into a falcon tube. For bare CNTs, sodium dodecyl sulfate was used to replace peptide. After the physical mixing process of the solution with sonication (5300, Ultrasonic Power Corporation, Freeport, IL, USA), the centrifuge separated the residual CNTs. The CNT solutions with P2 were diluted to 0.5 mg/mL and 0.1 mg/mL by adding more water (either 6.0 mL or 13.5 mL). The DEP process was used to assemble the peptide-functionalized CNTs on the chemiresistor. A total of 10 µL of the pipetted solution was placed across the electrode gaps, and an AC voltage (6 V_p-p_, 10 MHz) was applied. Depending on the target nanotube density, DEP time, and/or the level of self-limiting resistance (0 Ω, 100 Ω, or 1.2 KΩ) were changed. After rinsing with DI water and drying with house air, a peptide-functionalized CNT chemiresistor was prepared. Electrical resistance was checked for the CNT bundles formed across two electrodes. Assembled CNTs were observed and analyzed by a scanning electron microscope (SEM) (SU70, Hitachi, Tokyo, Japan) and atomic force microscope (AFM) (Dimension Icon, Bruker, Billerica, MA, USA).

### 2.4. Gas Exposure Setup and Measurement Protocols

An experimental setup used an acrylic chamber for the VOC exposure. The acrylic chamber included (1) an air inlet and outlet for the purging, (2) an IPA or acetone injection port, (3) an environmental (temperature and humidity) sensor, (4) an electric fan for facilitating the air circulation and VOC evaporation, and (5) the CNT chemiresistor clipped by an electrical connection jig. The semiconductor analyzer (Keithley 4200A-SCS, Tektronix, Beaverton, OR, USA) generated a constant current (1 µA), measured a voltage, and recorded measurement data over time. The semiconductor analyzer had a switching matrix (Keithley 708B, Tektronix, Beaverton, OR, USA) to multiplex eight pairs of electrodes (with a sampling rate of about 3 s). The measurement time was 1500 s (25 mg/L of VOC injection at 500 s and purging at 1000 s). When the VOC (IPA or acetone) was exposed, the concentration was 25 ppm. The voltage output was converted to the resistance values by dividing the voltage by the constant current. Baseline drift correction was performed by subtracting a linear fitting line (i.e., the least-squares regression) of the initial profile from 0–500 s.

## 3. Results and Discussion

### 3.1. Assembly of the Nanotube with Diverse Densities on the Chemiresistors

**Figure 1:**
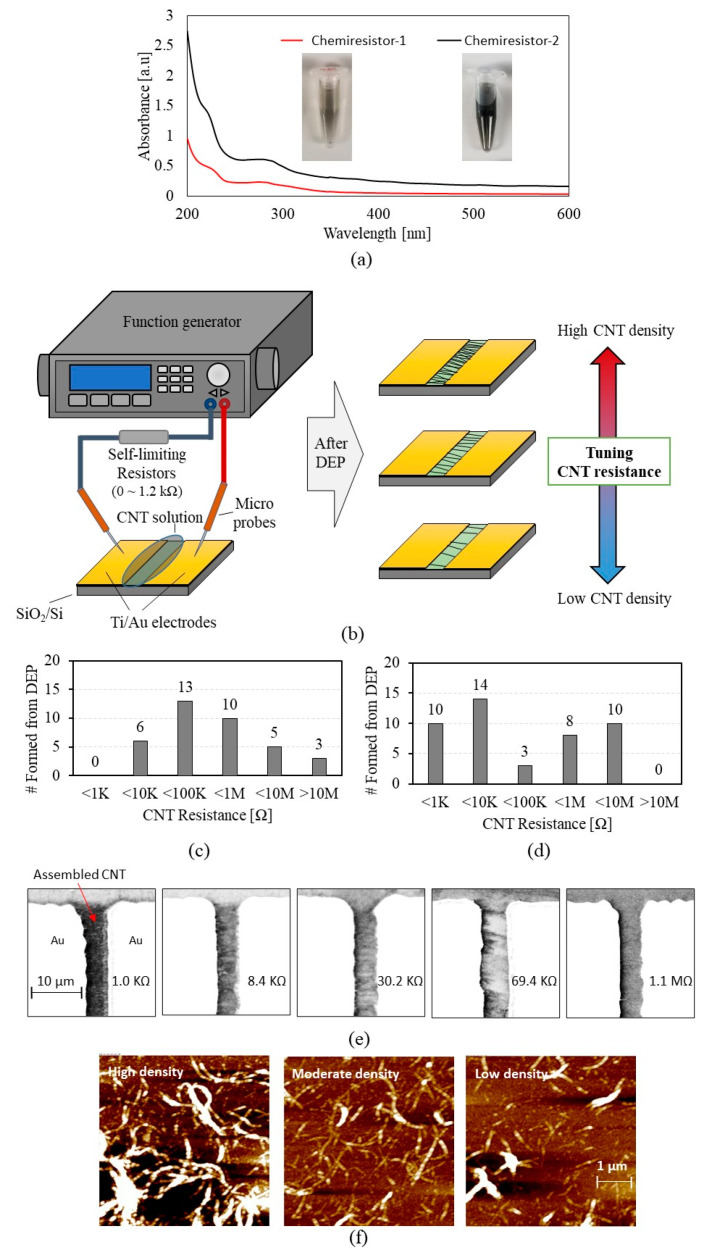
Peptide-functionalized carbon nanotube (CNT) assembly on the chemiresistor platform: (**a**) absorbance spectra of the peptide–CNT solutions for *chemiresistor 1* and *2*; (**b**) the schematics of the chemiresistor with dielectrophoresis (DEP) process where AC voltage is applied to assemble CNTs across the electrodes. DEP is controlled by the self-liming resistors (0~1.2 KΩ), DEP force, DEP time, and/or CNT solution concentration to yield the DEP-processed chemiresistors with different effectiveness of controlling the nanotube density densities; (**c**,**d**) the resistance ranges of the assembled CNTs for the *CNT chemiresistor-1* (*CCR1*) and *2* (*CCR2*), respectively; (**e**) the representative scanning electron microscope (SEM) images (×750); (**f**) the representative atomic force microscope (AFM) images showing different CNT resistance according to nanotube network density.

Figure 1 shows the preparation of peptide-functionalized CNT chemiresistors with varying nanotube densities. Two types of CNT chemiresistors, named *CCR1* and *CCR2*, were obtained from the CNT dispersions made with their respective in silico FYYYLLQ (P1) [32] and phage-displayed GSVQKLSATPWV (P2) [34] peptides. The preparation of the peptide-functionalized CNT solution is described in the Section 2. Figure 1a shows the absorbance spectra of the peptide-functionalized CNT solutions for *CCR1* and *2*, with pictures of the corresponding solutions. The peptide for *CCR2* shows a dispersion with a darker color, indicating higher CNT affinity than that of *CCR1*. Dielectrophoresis (DEP) was used to assemble CNT networks onto the chemiresistor platforms (Figure 1b). Several DEP variables were applied to make the various degree of CNT network devices. The assembly parameters included DEP force, DEP time, solution concentration, and resistance of self-limiting resistors. The detail of DEP conditions and results are indicated in Table 1. Briefly, CCR1 used three different fixed resistors (0 Ω, 100 Ω, and 1.2 KΩ), self-limiting resistors, serially connected to the device. The self-limiting resistor hinders the electric field applied to the device, thus resulting in a lower density of CNTs. The self-limiting resistors controlled CNT assembly to fabricate devices with various CNT densities. For CCR2, we used different solutions in CNT concentrations (1.0 mg/mL, 0.5 mg/mL, or 0.1 mg/mL) and different DEP times (0–60 s) to provide various devices with different CNT densities. We changed the DEP time only for bare CNT while keeping the self-limiting resistance of 0 Ω and CNT solution concentration of 1.0 mg/mL. Figure 1c,d shows the CNT resistance distributions for *CCR1* and *2*, respectively. In total, 37 *CCR1*, 45 *CCR2*, and 21 bare *CNT chemiresistors* were shown to vary their nanotube density and the corresponding resistance in the range of sub-KΩ to tens of MΩ. The peptide of *CCR1,* having a lower affinity to CNT than that of *CCR2*, results in a higher range of CNT resistance (3.43 KΩ–233 MΩ). The peptide of *CCR*2 has a higher affinity to CNT, resulting in a lower range of CNT resistance (0.48 KΩ–82.5 MΩ). Figure 1e,f shows the representative scanning electron microscope (SEM) and atomic force microscope (AFM) images of the CNT networks showing various nanotube density levels. 

Device-to-device variation inevitably occurs even if the same fabrication condition is used. This study utilizes the device-to-device variation to produce a variety of devices with various CNT densities. For example, the DEP condition with 6 Vp-p, 10 MHz, and 60 s results in CNT assembly with a range of 0.1–1 KΩ. If time is reduced to 10 sec, CNT assembly results in 0.5–100 KΩ. We repeated the DEP-based CNT assembly process to widely and evenly distributed devices with different nanotube densities. 

### 3.2. Characterization of Chemiresistor Signals

**Figure 2:**
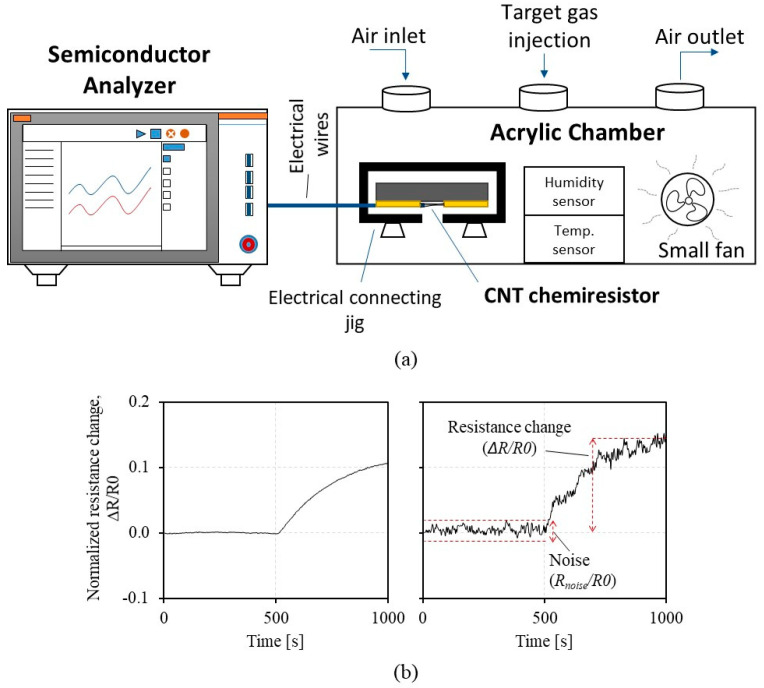
Experimental setup and representative signal profiles: (**a**) volatile organic compound (VOC) exposure testing, showing the semiconductor analyzer that measures and records resistance signals from chemiresistors, and the acrylic chamber composed of an electrical connecting jig containing a chemiresistor, a small fan, humidity/temperature sensors, air in/outlets, and a VOC injection hole; (**b**) responses from the *CCR1*s with the difference CNT resistances of 9.42 KΩ (left) and 164 KΩ (right), where isopropyl alcohol is exposed at 500 s, and signals are analyzed in terms of resistance change (Δ*R*), noise (*R_noise_*), and signal-to-noise ratio (Δ*R*/*R_noise_*) as shown in the middle graph in (**b**).

Figure 2a shows the experimental setup for the characterization of the chemiresistors. A more detailed setup and measurement protocol are described in the Section 2. Briefly, the prepared chemiresistor was placed in the electrical connecting jig inside the gas chamber. The semiconductor analyzer is connected to the jig to measure the voltage change in the chemiresistor continuously over time while applying the constant current bias. The VOC (either IPA or acetone) was injected into the chamber at 500 s, and the voltage measurement stopped at 1500 s. Figure 2b shows the representative resistance graphs from the *CCR1*s with a CNT resistance of 9.42 KΩ (high density) (left graph) and 164 KΩ (low density) (right graph). We used a sensitivity (normalized resistance change, Δ*R*/*R0*), a noise level (normalized noise, *R_noise_*/*R0*), and an SNR (Δ*R*/*R_noise_*) as the key parameters of the sensing characteristics following other gas sensor studies [35,36,37] that have accounted for these parameters to evaluate sensor performance. As shown in the right graph of Figure 2b, we define Δ*R*/*R0* as an amount of normalized resistance change (at 1000 s) in response to the exposed VOC. The *R_noise_*/*R0* is a root mean square average of the profile between 0–500 s when the signal zeros the baseline by subtracting the initial CNT resistance, *R0*. SNR is calculated from the Δ*R* divided by the *R_noise_*. The 9.42 KΩ *CCR1* (Figure 2b left) shows the notable Δ*R*/*R0* with a low *R_noise_*/*R0* to IPA exposure. The 164 KΩ *CCR1* (Figure 2b right) shows a higher Δ*R*/*R0*, but the *R_noise_*/*R0* is higher, too. These two profiles imply that CNTs with higher resistance (or lower density) may increase sensitivity and, at the same time, noise levels. We anticipate that (1) the tradeoff exists between sensitivity and noise level, and (2) the noise level significantly changes as CNT density (i.e., CNT resistance) changes.

### 3.3. Effect of Nanotube Density on IPA Sensing of CCR1s

**Figure 3:**
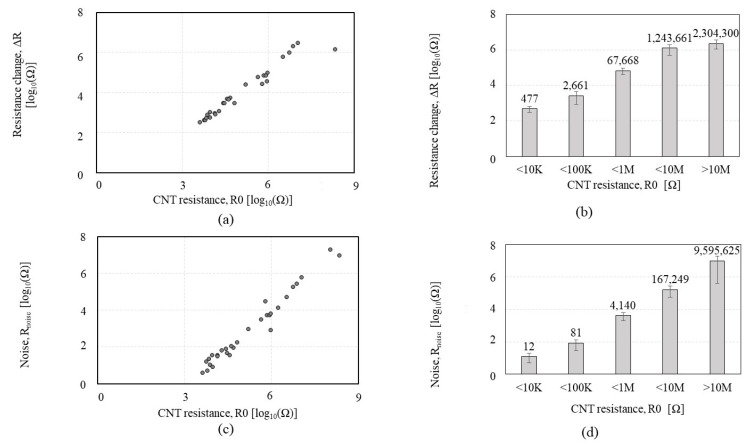
Sensing performances of the *CCR1* (*functionalized by* P1) depending on the CNT resistance, in response to isopropyl alcohol: (**a**,**b**) the resistance change (Δ*R*); (**c**,**d**) the noise (*R_noise_*).

To observe the effect of the nanotube density on the chemiresistor’s sensing performance and the tradeoff between sensitivity and noise level, we performed an IPA exposure test on 37 *CCR1*s with various CNT resistances (ranging from 3.43 KΩ to 233 MΩ). Figure 3 shows the Δ*R and R_noise_* of the *CCR1* depending on the CNT resistance in response to IPA exposure. It is observed that the Δ*R* increases as CNT resistance increases, as shown in Figure 3a,b. These indicate good agreement with the previous study [30], demonstrating that a higher CNT resistance shows a more significant response. The *R_noise_* (Figure 3c,d) shows the increasing noise level as CNT resistance increases. 

**Figure 4:**
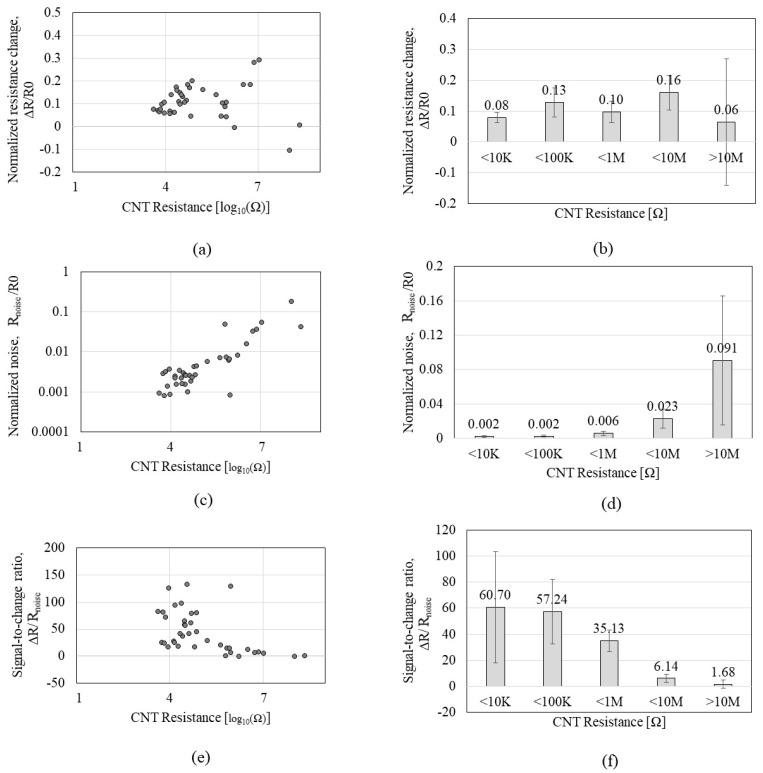
Sensing performance of the *CCR1* (*functionalized by* P1) depending on the CNT resistance, in a response to isopropyl alcohol: (**a**,**b**) the normalized resistance change (Δ*R*/*R0*); (**c**,**d**) the normalized noise (*R_noise_*/*R0*); (**e**,**f**) the signal-to-noise ratio (Δ*R*/*R_noise_*).

Then, we divided the Δ*R and R_noise_* with *R0* to normalize the data. Figure 4 shows the Δ*R*/*R0*, *R_noise_ /R0*, and Δ*R*/*R_noise_* of the *CCR1* depending on the CNT resistance in response to IPA exposure. As shown in Figure 4a,b, Δ*R/R0* tends to slightly increase as CNT resistance increases, but the significance of the increment is notably lower than Δ*R* in Figure 3a,b; the increment of Δ*R*/*R0* is negligible under the 1MΩ. When CNT resistance is more than 10 MΩ, the Δ*R*/*R0* shows a reduced response with a high standard deviation. *R_noise_*/*R0* (Figure 4c,d) tends to increase as CNT resistance increases, but it shows a plateau region in the range of 10–100 KΩ where *R_noise_*/*R0* level is minimum and constant. Figure 4e,f reveals that the CNT resistance in the 1–100 KΩ range showed the highest SNR. 

### 3.4. Effect of Nanotube Density on IPA Sensing of CCR2s

**Figure 5:**
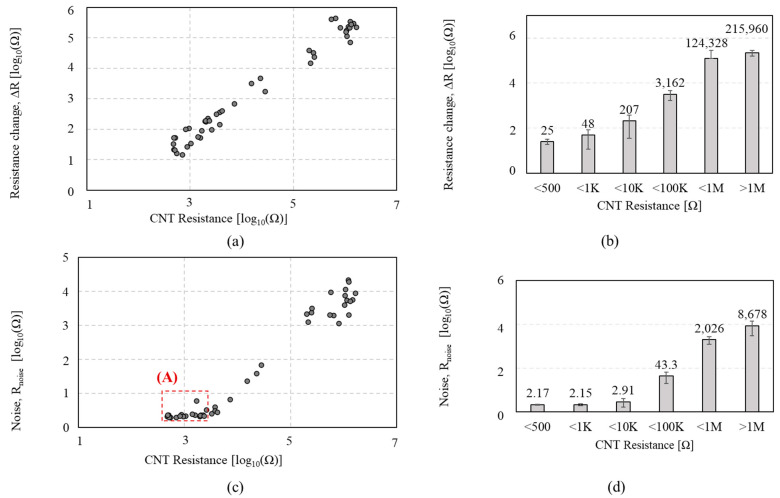
Sensing performances of the *CCR2* (*functionalized by* P2) depending on the CNT resistance in a response to isopropyl alcohol: (**a**,**b**) the resistance change (Δ*R*); (**c**,**d**) the noise (*R_noise_*). Red box A shows *R_noise_* stays static.

**Figure 6:**
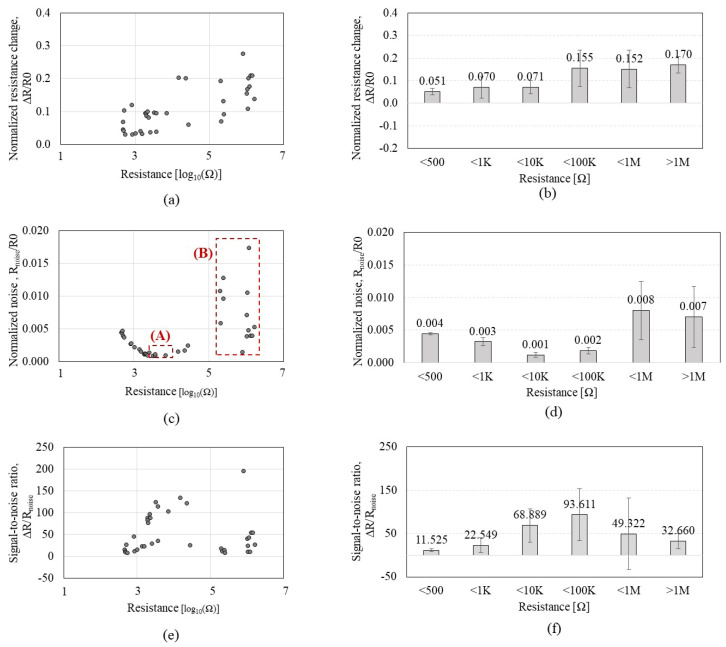
Sensing performances of the *CCR2* (*functionalized by* P2) depending on the CNT resistance in response to isopropyl alcohol: (**a**,**b**) the normalized resistance change (Δ*R*/*R0*); (**c**,**d**) the normalized noise (*R_noise_/R_0_*); (**e**,**f**) the signal-to-noise ratio (Δ*R*/*R_noise_*).

To further test sub-KΩ low-CNT resistance and to identify whether the tendency observed by P1 can be consistent with a different peptide, CNTs were functionalized by the P2 of GSVQKLSATPWV that exhibits a higher affinity to CNTs than P1. A total of 45 *CCR2*s with CNT resistances ranging from 0.48 KΩ to 82.5 MΩ were tested on the VOC gases. Figure 5 shows the Δ*R and R_noise_* of the *CCR2* in response to IPA exposure. Figure 5a,b reveals that Δ*R* increases as CNT resistance increases, which is a similar tendency to that of *CCR1*. Meanwhile, Figure 5c,d shows interesting results that reveal a plateau region in the range of 0–10 KΩ where *R_noise_* stays static (red box A in Figure 5c). Figure 6 shows the Δ*R*/*R0*, *R_noise_*/*R0*, and Δ*R*/*R_noise_* of the *CR2* depending on the CNT resistance in response to IPA exposure. As shown in Figure 6a,b, Δ*R*/*R0* tends to increase as CNT resistance increases, but the increment level is not significant, which is similar to that of *CCR1*; the increment of Δ*R*/*R0* is negligible under 10 KΩ. When CNT resistance is over 100 KΩ, the standard deviation is more than 50% of the average, which implies inconsistent CNT assembly. Regardless of this, many of MΩ-range CNT resistances did not respond to VOC exposure. *R_noise_*/*R0* (Figure 6c,d) indicates its minimum values (red box A in Figure 6c) in the resistance range of 1–10 KΩ. The second red box (B) in Figure 6c also indicates a high variation in *R_noise_*/*R0* when CNT resistance is over 1 MΩ. We anticipate that CNT resistance higher than 1 MΩ is likely to have an unstable network of CNT. High variation in *R_noise_*/*R0* also significantly affects SNR, as shown in Figure 6e,f. CNT resistances grouped as ‘<1 MΩ’ and ‘>1 MΩ’ show high standard deviations or low SNR, confirming a high possibility of having a weak tube-to-tube connection with a lack of SNR. Table 2 shows the standard deviation and coefficient of variation of each *CCR2,* where higher CNT resistance tends to show higher variations. We also observed that higher CNT resistance (>1 MΩ) shows a 50% lower yield rate than lower CNT resistance (<10 KΩ) during the DEP-based assembly process. CNT resistance in the range of 1–10 KΩ is the suitable level for the chemiresistors in terms of SNR, fabrication yield rate, and stability, which aligns with the results from the *CCR1* test.

### 3.5. Effect of Nanotube Density on Acetone Sensing of CCR2

**Figure 7:**
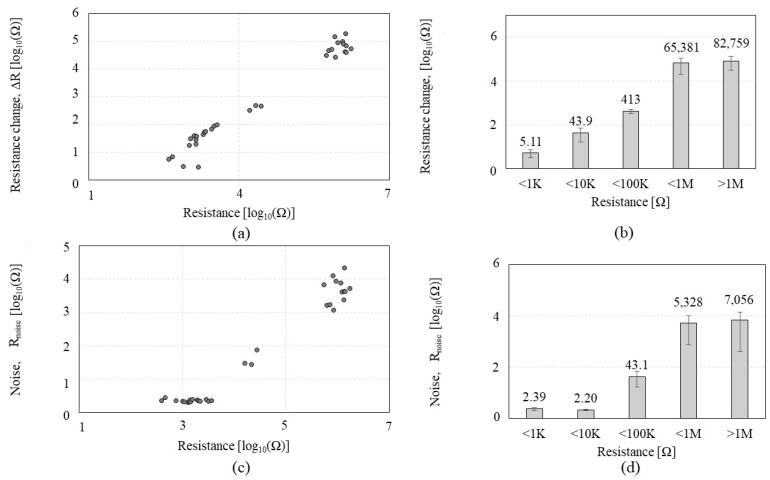
Sensing performances of the *CCR2* (*functionalized by* P2) depending on the CNT resistance in a response to acetone: (**a**,**b**) the resistance change (Δ*R*); (**c**,**d**) the noise (*R_noise_*).

**Figure 8:**
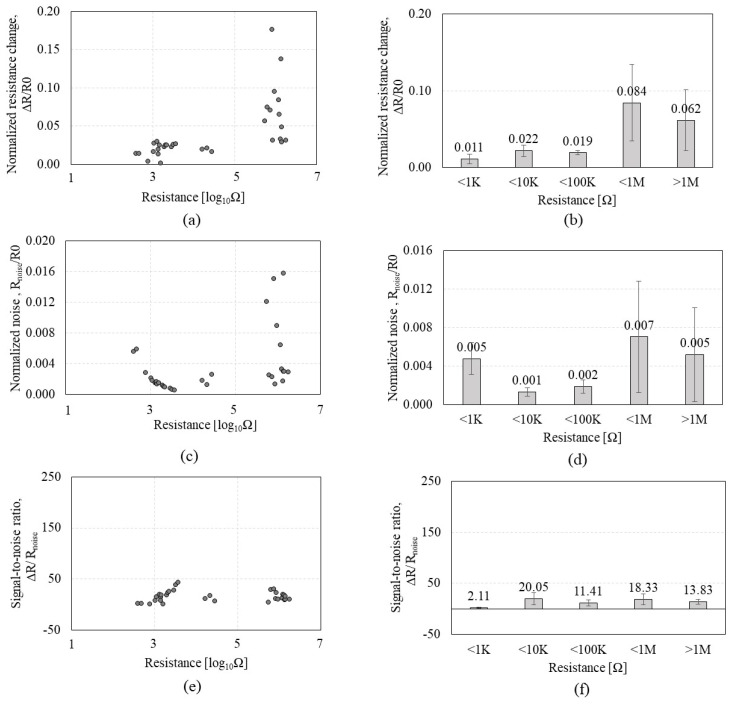
Sensing performances of the *CCR2* (*functionalized by* P2) depending on the CNT resistance in a response to acetone: (**a**,**b**) the resistance change ratio (Δ*R*/*R0*); (**c**,**d**) the noise ratio (*R_noise_*/*R0*); (**e**,**f**) the signal-to-noise ratio (Δ*R*/*R_noise_*).

*CCR2s* were also exposed to acetone vapors to examine whether the nanotube density optimized to IPA is expandable to another VOC. Figure 7 shows the Δ*R and R_noise_* of the *CCR2* from the same set of *CCR2s* in response to acetone exposure. Here, the Δ*R* increases as CNT resistance increases (Figure 7a,b), showing a similar tendency to IPA exposure tests for the *CCR1* and *2*. The *R_noise_* also increases as CNT resistance increases (Figure 7c,d). There is also the plateau where noise level is static in the range of <10 KΩ, similar to the IPA exposure test. Figure 8 shows the Δ*R/R0*, *R_noise_*/*R0*, and Δ*R*/*R_noise_* of the *CCR2* depending on the CNT resistance in response to acetone exposure. The Δ*R*/*R0* and *R_noise_/R0 are* both high standard deviations when CNT resistance is more than 1 MΩ. In the less than 1 MΩ range, the Δ*R/R0 and R_noise_/R0* in 1–10 KΩ showed maximum and minimum values, respectively. CNT resistance in the range of 1–10 KΩ shows the highest SNR (Figure 8e,f), aligning with the data in Figure 3, Figure 4, Figure 5 and Figure 6. The SNR of acetone is not as high as that of IPA since the functionalized peptide does not attract acetone as much as IPA. This acetone test confirms that the effect of nanotube density observed from IPA vapors can be expandable to another VOC. 

**Figure 9:**
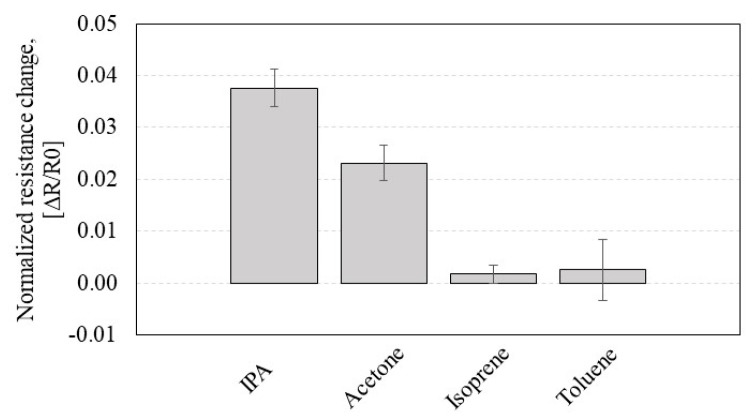
Normalized resistance change (Δ*R*/*R0*) of the *CCR2* (*functionalized by* P2) in response to four different VOCs for the selectivity test, where the resistance of the CNT ranges 500 Ω–10 KΩ.

In the IPA vs. acetone test, the CCR2 device clearly shows higher response to IPA than to acetone. We investigated further the selectivity of the CNT sensors using two more VOCs (Figure 9). The normalized resistance change (Δ*R/R0*) in response to four different VOCs (IPA, acetone, isoprene, and toluene) shows that the exposure to IPA has the highest response while the comparable isoprene and toluene exposures did not show noticeable resistance changes. The mechanism is presumed to be the hydroxyl group of IPA forming hydrogen bonds with threonine or serine in the P2 sequence, thus resulting in the significant Δ*R*/*R0*. On the other hand, CCR2 is less responsive to acetone and shows negligible responses to isoprene and toluene, which are non-polar VOCs. Additional tests with in-depth analysis tools (e.g., molecular dynamic simulations, microarrays, etc.) will be used in future work to investigate the VOC sensing mechanism further.

### 3.6. Effect of Nanotube Density on IPA Sensing of Bare CNT Chemiresistors

**Figure 10:**
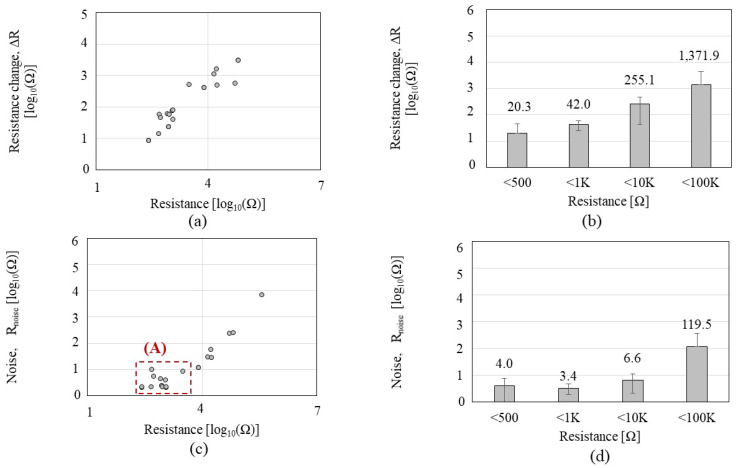
Sensing performance of the bare *CNT chemiresistor* depending on the CNT resistance in response to IPA: (**a**,**b**) the resistance change ratio (Δ*R*); (**c**,**d**) the noise ratio (*R_noise_*).

**Figure 11:**
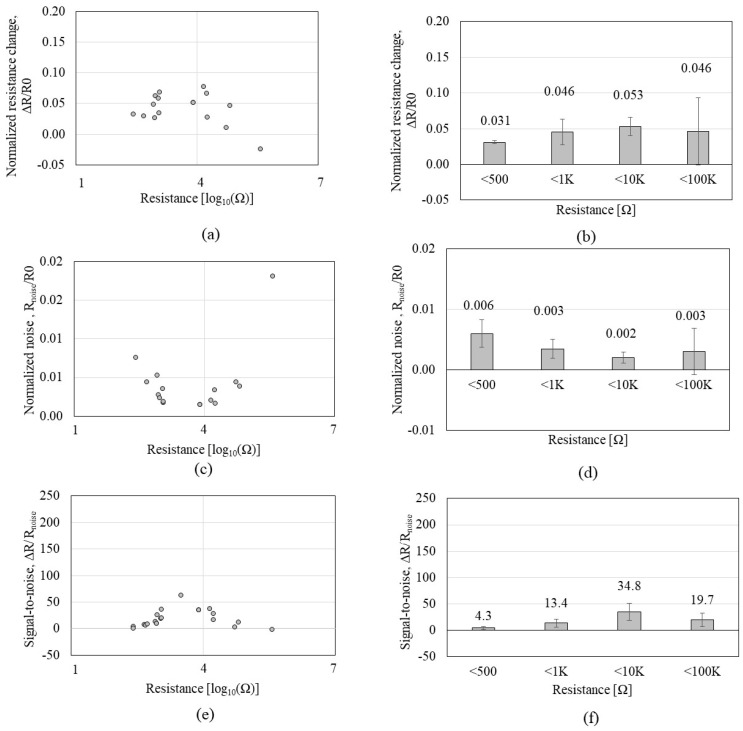
Sensing performances of the bare *CNT chemiresistor* depending on the CNT resistance in response to IPA: (**a**,**b**) the resistance change ratio (Δ*R*/*R0*); (**c**,**d**) the noise ratio (*R_noise_*/*R0*); (**e**,**f**) the signal-to-noise ratio (Δ*R*/*R_noise_*).

We also prepared bare *CNT chemiresistors* (21 total) without peptide functionalization. We exposed IPA vapors to investigate the effect of the functionalized peptide on CNT sensor responses to VOC. Figure 10 shows the Δ*R* and *R_noise_* of the bare *CNT chemiresistor* in response to IPA exposure. Here the Δ*R* increases as CNT resistance increases (Figure 10a,b), showing a similar tendency with IPA exposure tests for the *CCR1* and *2*. The *R_noise_* also increases as CNT resistance increases (Figure 10c,d). Unlike peptide-based chemiresistors, no plateau is observed in the <10 KΩ (red box A in Figure 10c) range. Figure 11 shows the Δ*R*/*R0*, *R_noise_*/*R0*, and Δ*R*/*R_noise_* of the bare *CNT chemiresistor* in response to IPA exposure. As a peptide-based *CCR*2, the *R_noise_*/*R0* showed minimum values in the <10 KΩ range. Figure 11e,f shows the SNR depending on the CNT resistance, where CNT resistance of 1–10 KΩ shows the highest value. The sensitivity and SNR of bare CNT chemiresistors are lower than that of peptide-functionalized CNTs. The noise level is relatively higher (about 2 times) than that of the peptide-functionalized CNTs. The bare CNT test indicates that the functionalized peptide does serve as a CNT stabilizer as well as a significant sensing element for VOC detection. We found that the CNT resistance of 1–10 KΩ is observed to be optimal for the VOC sensing with moderately low-resistance CNT chemiresistors, for a set of defined electrodes with 100 µm width and 5 µm gap.

Overall, based on the results from three types of chemiresistors (*CCR*1 and 2 and bare CNT chemiresistors) exposed to two chemicals, IPA and acetone, Δ*R* and *R_noise_* consistently increase as CNT resistance increases. When the data is normalized, Δ*R*/*R0* still tends to increase, but its significance is low and negligible at the CNT resistance level under 100 KΩ. *R_noise_*/*R0* shows its minimum point, typically in the 1–10 KΩ range. The standard deviations of the sensing performance (sensitivity and noise level) significantly increase when CNT resistance is higher than 1 MΩ. Considering these observed factors, the CNT resistance range for the best SNR can be optimized. When we tested further by changing either the VOC from IPA to acetone or the peptide-functionalized CNT to bare CNT, they showed a consistent tendency but lower sensitivity and SNR levels. 

### 3.7. SEM Investigations for Mechanism Analysis

**Figure 12:**
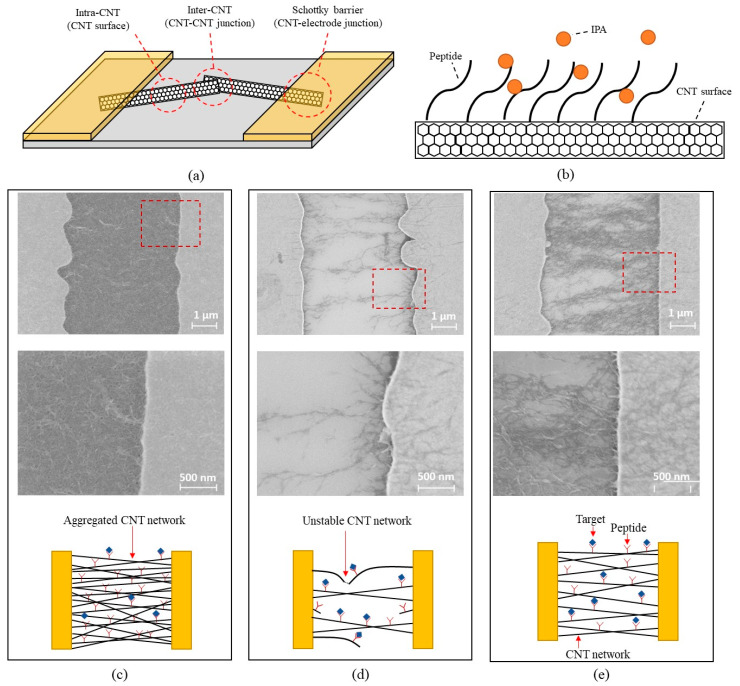
The mechanism of the effect of nanotube density: (**a**) 3 dominant factors related to CNT sensors; (**b**) the schematics showing events between IPA and peptides on the CNT surface; (**c**–**e**) the scanning electron microscope images and schematics with the example of the aggregated CNT network reducing the event area and efficiency (**c**), the lack of the CNT network causing the unstable transduction of the events (**d**), and the appropriate density of the CNT network (**e**).

The network of CNTs is associated with some parameters that affect sensing mechanisms. It has been reported that sensing mechanisms of the CNT sensor are related to intra-CNT properties (CNT surface), inter-CNT network (CNT-CNT junction), and the Schottky barrier (CNT–electrode junction) [38], as shown in Figure 12a. There has still been controversy regarding which factor among the three regions is dominant for CNT sensors [29,39,40]. Salehi-Khojin et al. [41] suggested that the defect level of the CNT could change the dominant sensing mechanism. Perfect CNT networks showed high relevance to the Schottky barrier for molecule sensing, whereas a defective CNT network was more related to the intra-CNT effect. Boyd et al. [42] insisted that the CNT network density changed a dominant contributor to the sensing mechanism. The higher density of CNTs was mainly affected by intra-CNT properties, while the lower density of CNTs showed high dominance of the Schottky barrier. These studies imply that the dominant sensing mechanism can differ depending on the circumstances of the CNTs, internally and externally configured. BREs serve as the sensing materials on the CNT surface and attract the target molecule, as schematically demonstrated in Figure 12b. Several previous CNT sensors have demonstrated that BRE-functionalized CNTs [21,43,44,45] exhibited selective and sensitive characteristics, where computational predictions and experimental results have proved the interface between BREs and target molecules. In this regard, we presume that the sensing mechanism of the BRE-functionalized CNT network is dominantly associated with the intra-CNT effect. Figure 11c–e explains the principles of the experimentally observed trends. The lower resistance CNT has a lower sensitivity because of the screening effect due to the CNT aggregation, as shown in Figure 12c. The screening effect causes a reduction in the surface area of the CNTs, decreasing the possibility for BREs to interact with target molecules. Therefore, a less dense CNT network with high resistance can facilitate events between BREs and target molecules. However, high CNT resistance causes an unstable network resulting in a higher noise level and device-to-device variation, as indicated in Figure 12d, where the image reveals that some CNT strands are disconnected. The experimental data exhibited that the noise level and device-to-device variation more significantly impact SNR than sensitivity. Therefore, it is desirable to have a moderately low CNT resistance to alleviate an unstable nanotube network and obtain the best SNR. Figure 12e shows the example of the optimal CNT network configuration showing both high surface area and a robust network that guarantees secure electrical contact. This appropriate network condition (in the range of 1–100 KΩ CNT resistances for the given chemiresistor platform) resulted in the best SNR. The summary of the results is shown in Table 3.

## 4. Conclusions

This study revealed the effectiveness of the nanotube density and its working principle as a crucial design factor for peptide-functionalized CNT chemiresistors. We explored the effect of CNT density by using three types of representative chemiresistors with the following: (a) CNT functionalized by the peptide from a computation methodology (*CCR1*), (b) CNT functionalized by the peptide derived from a biopanning methodology (*CCR2*), and (c) bare CNT with SDS surfactant (bare *CNT chemiresistor*). We utilized the DEP process to build diverse CNT densities for the three representative chemiresistors. As a result, we proposed the general finding that noise level and device-to-device variation play essential roles in determining the optimal SNR as CNT density changes. This study demonstrates the extensive experimental analysis of the effect of nanotube density, successfully providing an essential criterion for designing BRE-based CNT chemiresistors.

## Figures and Tables

**Table 1:** Dielectrophoresis (DEP) conditions and results for the carbon nanotube (*CNT*) *chemiresistors 1* and *2* that are functionalized by different peptides.

		*CNT Chemiresistor-1* *(CCR1)*	*CNT Chemiresistor-2* *(CCR2)*	Bare *CNT Chemiresistor*
Functionalized peptide sequence		FYYYLLQ	GSVQKLSATPWV	-
DEP Conditions (to tune CNT resistance)	Self-limiting resistance	0 Ω, 100 Ω, 1.2 KΩ	0 Ω	0 Ω
	CNT–peptide solution concentration	1.0 mg/mL	1.0 mg/mL, 0.5 mg/mL, 0.1 mg/mL	1.0 mg/mL
	DEP time	60 s	0–60 s	0–60 s
	DEP force	6 V_p-p_, 10 MHz		
DEP results	# of devices with CNT assembled	37 (out of 51)	45 (out of 56)	21 (out of 24)
	Resistance range of CNT assembled	3.43 KΩ–233 MΩ	0.48 KΩ–82.5 MΩ	0.26–372 KΩ

**Table 2:** Standard deviation and coefficient of variation (CV) for the signal-to-noise ratio (SNR) of the carbon nanotube (*CNT*) *chemiresistors*-*2* depending on the CNT resistance level.

CNT Resistance Range (Ω)	<500 Ω	<1 KΩ	<10 KΩ	<100 KΩ	<1 MΩ	>1 MΩ
SNR Standard deviation (Ω)	3.40	16.89	38.41	59.75	82.05	17.63
SNR CV (%)	29.50	74.91	55.76	63.83	166.36	53.99

**Table 3:** Summary of the present study in comparison with previous works.

	Characterized Sensing Performance	CNT Types	Tested Devices #	Target to Detect	Results (Findings)
Ref. [29]	Sensitivity Detection limit	Bare SWCNT	3	Streptavidin	Lower density offers higher sensitivity
Ref. [30]	Sensitivity	Bare SWCNT Bare MWCNT	8	NO_2_	Density proportionally relates to senstivity
Present work	Sensitivity Noise SNR	Peptide-SWCNT Bare SWCNT	103	IPA Acetone	Moderately high density is desirable for the optimized SNR

SWCNT: single-walled CNT, MWCNT: multi-walled CNT.

## Data Availability

The data presented in this study are available on request from the corresponding author.
